# Characteristic and Prognostic Implication of Venous Thromboembolism in Ovarian Clear Cell Carcinoma: A 12-Year Retrospective Study

**DOI:** 10.1371/journal.pone.0121818

**Published:** 2015-03-20

**Authors:** Shuang Ye, Jiaxin Yang, Dongyan Cao, Huimin Bai, Huifang Huang, Ming Wu, Jie Chen, Yan You, Jinghe Lang, Keng Shen

**Affiliations:** 1 Department of Obstetrics and Gynecology, Peking Union Medical College Hospital, Chinese Academy of Medical Sciences & Peking Union Medical College, Beijing, China; 2 Department of Pathology, Peking Union Medical College Hospital, Chinese Academy of Medical Sciences & Peking Union Medical College, Beijing, China; University of North Carolina School of Medicine, UNITED STATES

## Abstract

**Purpose:**

To profile the characteristic and prognostic implications of venous thromboembolism (VTE) in Chinese ovarian clear cell carcinoma (CCC) patients.

**Methods:**

We identified all of the cases between 2000 and 2012 by searching our institutional Ovarian CCC Database. A comprehensive review of the medical documentation was performed to collect relevant data. Kaplan-Meier models and Cox regression were employed for survival analysis.

**Results:**

Of the 227 patients, 33 (14.5%) experienced VTE events. There was no significant difference between VTE and non-VTE group patients regarding age, serum cancer antigen 125 or tumor size. The optimal cytoreduction rate was higher in patients without VTE (70.1%) than in those with VTE (51.5%). VTE events were more likely to occur at presentation (36.4%) and recurrence (33.3%), followed by an adjuvant chemotherapy period (18.2%). VTE was more common in patients with advanced-stage disease than those with early-stage disease (P=0.003), whereas pulmonary embolism (PE) was 10-fold as common in advanced-stage disease as in early-stage disease (8.6% vs. 0.8%, P = 0.012). Patients with advanced disease tended to have thrombi in the proximal veins. Two patients died of PE, as confirmed by autopsy. Patients with VTE had reduced survival compared to those without VTE (median overall survival 54 vs. 140 months, P<0.001; median progression-free survival 17 vs. 43 months, P<0.001).

**Conclusions:**

Overall, 14.5% of the patients with ovarian CCC experienced VTE, mainly before their cancer diagnosis or at a time of recurrence. VTE adversely impacted patient survival.

## Introduction

Venous thromboembolism (VTE), comprising deep vein thrombosis (DVT) and pulmonary embolism (PE), is one of the leading causes of death in patients with active cancer [1,2]. Women with gynecologic cancers are at high risk for VTE [3] due to their intrinsic malignancy, advanced stages, pelvic masses and lengthy abdominal and pelvic operations [4]. This feature is especially true for ovarian cancer patients who present with advanced-stage disease and receive complicated treatment regimens. A large population-based study showed that, among 13,031 cases of ovarian cancer, 5.2% were diagnosed with a VTE event within 24 months after diagnosis [5]. Ovarian clear cell carcinoma (CCC) is considered a distinct histological subtype with a high frequency of VTE [6,7].

The prognostic implications of VTE in cancer have been studied in different types of cancer [5–9]. Ever-increasing data suggest that VTE in ovarian CCC is associated with tumor aggressiveness and can adversely impact the survival of patients [6,7]. Several important studies have been undertaken to investigate the associations between VTE and ovarian CCC in different groups of people [6,7,10,11]. As is generally accepted, a higher incidence of ovarian CCC was found among Asian women [12,13]. However, none of the published studies focused on the association between VTE and ovarian CCC in Chinese patients, even though China is a high-frequency region regarding ovarian CCC.

Thus, the present study aimed to assess the incidence and characteristics of VTE events in Chinese patients with ovarian CCC based on our own data and to investigate the possible prognostic implications of VTE in ovarian CCC.

## Materials and Methods

### Study Subjects

This study was approved by the Institutional Ethical Committee of Peking Union Medical College Hospital. The Ovarian Clear Cell Carcinoma database was set up and is maintained by research faculty members in our department. All of the CCC patients diagnosed and treated in our hospital since 1982 were included, and their basic information was recorded in the database. Using the database, we identified all patients between the years 2000 and 2012. The patients included in this study had to fulfill the following criteria: 1) ovarian CCC as a histologic diagnosis by pathologists via pathology reports in the medical chart and microscopic slides reviewed by a single experienced gynecologic pathologist (Dr. You); and 2) management and follow-up at our institution. All patients gave their written informed consent prior to inclusion in the study.

### Data Collection

A comprehensive review of the medical charts was performed to collect the data listed as follows: age at diagnosis, serum cancer antigen 125 (CA 125) level, primary surgery date and type, ascites volume, residual disease, primary tumor size, International Federation of Gynecology and Obstetrics (FIGO) stage, lymph node dissection, adjuvant chemotherapy, the presence of VTE, date of disease progression or recurrence and disease status at last contact. Unfortunately, the above information was not all available in some cases, such as preoperative CA 125 level (n = 30) and tumor size (n = 37).

All patients were staged by the FIGO 1999 staging system, according to the CRS and pathological findings. In our institution, patients at stage I and II underwent complete staging surgery, and patients at stage III and IV received cytoreductive surgery (CRS) as a standard approach. Optimal CRS was defined as residual disease less than 1 cm after a surgical procedure. The primary tumor size was measured by gynecologic oncologists during the operation and recorded in the surgical note. Adjuvant chemotherapy was routinely administered after primary surgery (usually within 14 days). In the study period, all patients received a platinum-based chemotherapy regimen. The normal upper limit of serum CA 125 was 35 U/ml. In this study, the post-operative period was defined as the interval from surgery to the first cycle of chemotherapy.

Progression-free survival (PFS) was calculated as the time interval from primary surgery to the date of disease progression or recurrence. Overall survival (OS) was defined as time interval from the date of the primary surgery to the date of death or last contact.

### Diagnosis and prophylactic treatment of VTE

The diagnosis and prophylactic treatment of VTE was based on clinical impressions and imaging findings. Patients with suspicious complaints were submitted to further imaging modalities to confirm the diagnosis. DVT was diagnosed by Doppler Ultrasound and Computerized Tomography; PE, by spiral computerized tomography pulmonary angiogram (CTPA) and ventilation-perfusion lung scan. The proximal veins included the inferior vena cava (IVC), iliac veins, femoral veins and popliteal veins, whereas the distal veins included the great saphenous, peroneal, posttibial and soleal veins [14]. DVT cases involving both proximal and distal veins were classified into proximal cases. All patients with gynecologic malignancies were considered at high risk for VTE and were asked to wear Thromboembolic Deterrent (TED) stockings postoperatively. Each of these patients was treated with prophylactic anticoagulation therapy (low molecular weight heparin) from 48 hours after surgery to 14 days.

### Statistical analyses

Statistical analyses were performed using SPSS statistical software (Version 17.0, SPSS, Inc, Chicago, IL) and GraphPad Prism (Version 5.0, GraphPad Software, Inc, La Jolla, CA). The chi-square and parametric Student *t* tests were employed to compare the differences between the two groups (ovarian CCC with or without VTE). Survival time was calculated using the Kaplan-Meier model, whereas Cox regression was performed for multivariate analysis. Variables with statistical significance on univariate analyses were included in the multivariate ones. All of the *P* values reported were two-tailed, and statistical significance was set at a *P* value of <0.05.

## Results

During the study period from 2000 to 2012, 227 CCC patients fulfilled the inclusion criteria. Of these, 33 (14.5%) patients developed documented VTE, whereas 194 (85.5%) patients had no evidence of VTE. Patients with confirmed VTE were treated immediately by standard anticoagulation therapy. IVC filters were placed in some cases to prevent a lethal PE in patients with thrombus in the proximal veins. The clinical and pathological characteristics of ovarian CCC patients with or without VTE are summarized in Table 1.

**Table 1:** Clinicopathological features of ovarian CCC patients with or without VTE.

Variables	Cohort	VTE group	Non VTE group	*P* *
Number	227(100%)	33(14.5%)	194(85.5%)	
Age (range) (years)	50(29–81)	51(31–73)	49(29–81)	0.379
Serum CA 125 (Mean ± SD) (U/ml)	811.5±1681.9 n = 197	960.6±2409.9 n = 25	789.7±1556.8 n = 172	0.734
Stage Group				
Early (I+II)	122(53.7%)	10(30.3%)	112(57.7%)	0.003
Advanced (III+IV)	105(46.3%)	23(69.7%)	82(42.3%)	
Primary Tumor size (Mean ± SD) (range) (cm)	10.9±5.11(1–30) n = 190	12.1±5.58(3–25) n = 28	10.6±5.01(1–30) n = 162	0.213
Primary tumor size≥ 10cm	98(51.6%)	16(57.1%)	82 (50.6%)	0.523
Ascites ≥0.5L	51(22.5%)	10(30.3%)	41(21.1%)	0.243
Residual disease				
≤1cm (optimal)	153(67.4%)	17(51.5%)	136(70.1%)	0.035
>1cm (suboptimal)	74(32.6%)	16(48.5%)	58(29.9%)	
Lymph node dissection	196(86.3%)	21(63.6%)	175(90.2%)	<0.001
Adjuvant chemotherapy	219(96.4%)	31(93.9%)	188(96.9%)	0.731
Status				
Alive	154(67.8%)	12(36.4%)	142(73.2%)	<0.001
Dead	73(32.2%)	21(63.6%)	52(26.8%)	

*Chi-square test and parametric student *t* test.

Abbreviations: CCC, clear cell carcinoma; SD, standard deviation; VTE, venous thromboembolism; CA 125, cancer antigen 125.

### Clinical and pathological features

In general, the mean age of the patients in this series was 50 years (range, 29–81 years), and 53.7% (122/227) of the patients were diagnosed at early-stage (FIGO I+II) disease. Nearly a quarter (22.5%, 51/227) of the patients presented with a large volume of ascites (more than 0.5 L). The mean tumor size measured by the operating surgeons was 11 cm (range, 1–30 cm). Optimal cytoreduction was achieved in 67.4% (153/227) of the cases. Lymphadenectomy was performed in 86.3% (196/227) of the patients, whereas 96.4% (219/227) of the patients received adjuvant chemotherapy. For the study period, we observed a total of 73 deaths. As can be clearly seen in Table 1, there were no significant differences between the two groups of patients (with or without VTE) regarding age, CA 125 level, tumor size or ascites. There were statistically significant differences in the tumor stage, residual disease, lymphadenectomy and current status. Patients with VTE were more likely to have advanced disease compared to those without VTE (69.7% vs. 42.3%, *P* = 0.003). Optimal debulking was achieved in more patients without VTE than in those with VTE (70.1% vs. 51.5%, *P* = 0.035). More patients in the non-VTE group underwent lymph node dissections (*P*<0.001).

**Fig 1:**
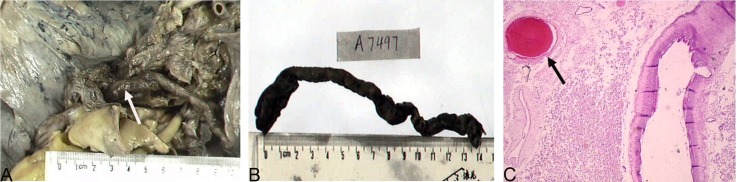
Pulmonary emboli in both pulmonary arteries, as demonstrated by autopsy in a patient who died of pulmonary embolism. Please refer to the main text for the details of the case. Fig. 1a macroscopic thrombus (arrows); Fig. 1b a 20-cm long thrombus; Fig. 1c microscopic thrombus (arrows), hematoxylin and eosin stain, magnification X100.

The features of VTE in ovarian CCC, including timing and type of VTE, are shown in Table 2. Regarding the time pattern, twelve (36.4%) patients had VTE as an initial presentation before their cancer diagnosis; four (12.1%) patients were diagnosed in the post-operative period; six (18.2%) patients developed VTE during the chemotherapy; and eleven (33.3%) patients had VTE at the time of tumor progression or recurrence. Of the twelve patients with VTE diagnosed before surgery, five (41.7%) patients presented simultaneously with both PE and DVT; six (50.0%) patients had DVT alone (four patients in proximal veins); and one (8.3%) patient had PE alone. Seven (58.3%) patients (five patients with both PE and DVT and two patients with DVT) underwent the placement of IVC filters prior to their primary surgery. The median interval between VTE and diagnosis of ovarian carcinoma (date of surgery) was 38 days (range, 2–120days). All post-operative DVT were detected because of complaints of leg swelling or pain, on days 6 or 7 post-operative. During chemotherapy, four patients developed DVT, and two had PE. Unfortunately, these two patients (6.1%) experienced sudden death due to PE, as confirmed by autopsy. One patient, who was also reported at our clinicopathological conference, experienced left-sided hemiparalysis after the first cycle of chemotherapy [15]. This finding was confirmed by computerized tomography to be cerebral ischemic infarction. Three months later (third cycle of chemotherapy), the patient died of a PE resulting from a 20-cm long thrombus in both pulmonary arteries (shown in Fig. 1). Of the patients with VTE diagnosed at the time of tumor progression or recurrence, 81.8% (9/11) of the patients had DVT alone; only two patients (18.2%) had simultaneous PE and DVT. IVC filters were placed in three patients (two with DVT and one with both DVT and PE) before secondary CRS.

**Table 2:** Features of venous thromboembolism (VTE) in ovarian clear cell carcinoma.

	DVT alone	PE+DVT	PE alone	Total number (%)
Before operation	6	5	1	12 (36.4%)
After operation	4	0	0	4 (12.1%)
During chemotherapy	4	0	2	6 (18.2%)
At recurrence	9	2	0	11 (33.3%)
Total number (%)	23 (69.7%)	7 (21.2%)	3 (9.1%)	33(100%)

Abbreviations: VTE, venous thromboembolism; DVT, deep vein thrombosis; PE, pulmonary embolism.

Table 3 further illustrates the distribution of patients by FIGO stage and VTE type. VTE was more commonly reported in advanced-stage disease (23/105, 21.9%) than in early-stage disease (10/122, 8.2%) with statistical significance (*P* = 0.003, Pearson test). In addition, patients with advanced disease tended to have thrombus in the proximal veins (7/105, 6.6%), compared to patients with early disease (2/122, 1.6%), although statistical significance was not achieved (*P* = 0.085, Fisher exact test). PE was 10-fold as common in advanced-stage disease (9/105, 8.6%) as in early-stage disease (1/122, 0.8%), which was statistically significant (*P* = 0.012, Fisher exact test).

**Table 3:** Distribution of patients with ovarian clear cell carcinoma by stage and number of VTE.

Stage	Patients (%)	VTE events					
		DVT alone		PE+DVT	PE alone	Total VTE (% per stage)	%Total VTE
		Proximal	Distal				
I	107(47.1%)	2	5	1	0	8(7.5%)	24.2%
II	15(6.6%)	0	2	0	0	2(13.3%)	6.1%
III	93(41.0%)	5	6	6	3	20(21.5%)	60.6%
IV	12(5.3%)	2	1	0	0	3(25.0%)	9.1%

Abbreviations: VTE, venous thromboembolism; DVT, deep vein thrombosis; PE, pulmonary embolism.

### Survival analysis

The mean follow-up time was 50 months (range, 1–153 months) for the entire cohort. As mentioned above, two (6.1%) out of 33 patients died directly due to VTE-related complications (fatal PE developed in 4.5 and 1 months after surgery).

**Fig 2:**
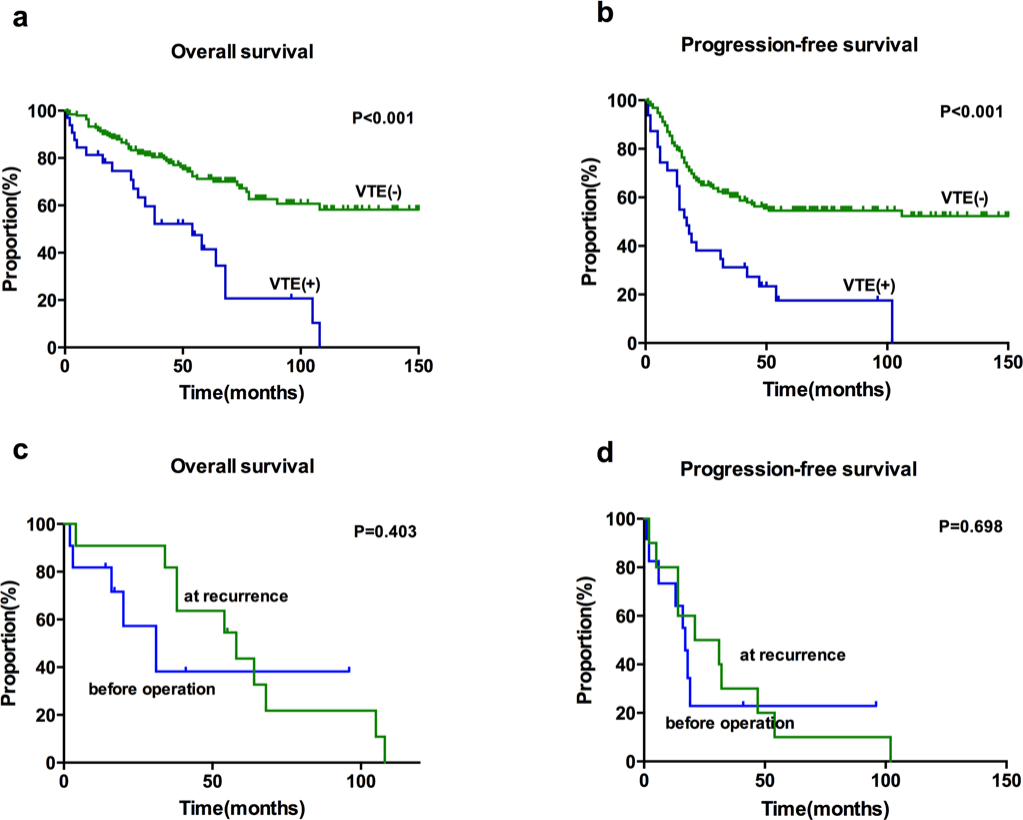
Kaplan-Meier survival curves of venous thromboembolism (VTE) in ovarian CCC. (a). Overall survival analyzed for cases with or without VTE. (b). Progression-free survival analyzed for cases with or without VTE. (c) Overall survival analysis based on timing of VTE. (d) Progression-free survival analysis based on the timing of the VTE.

Patients who experienced VTE had worse survival outcomes, with a shorter median OS and PFS compared to patients without VTE (median OS 54 vs. 140 months, *P*<0.001, [Fig. 2a]; median PFS 17 vs. 43 months, *P*<0.001, [Fig. 2b]). Further subgroup analysis based on VTE timing revealed that there was no significant difference between patients who developed VTE at presentation and those at recurrence (Fig. 2. C-D). The type of VTE was not associated with OS (DVT alone vs. DVT+PE, *P* = 0.566) or PFS (DVT alone vs. DVT+PE, *P* = 0.555).

Table 4 is the result of univariate and multivariate survival analysis. After controlling for stage and residual disease, VTE remained a statistically significant adverse variable for OS and PFS.

**Table 4:** Significant predictors of survival in univariate and multivariate survival analysis.

UNIVARIATE ANALYSIS					
Variable	Category	No.	Percentage	*P* (OS)	*P* (PFS)
Age (years)	<50	119	52.4%	0.383	0.987
	≥50	108	47.6%		
Stage	Early	122	53.7%	<0.001	<0.001
	Advanced	105	46.3%		
Residual disease	Optimal (<1cm)	153	67.4%	<0.001	<0.001
	Suboptimal (≥1cm)	74	32.6%		
VTE	Yes	33	14.5%	<0.001	<0.001
	No	194	85.5%		

MULTIVARIATE ANALYSIS						
Variable	Overall survival			Progression-free survival		
	HR	95% CI	*P*	HR	95% CI	*P*
Stage	1.721	0.853–3.474	0.130	2.677	2.606–5.781	0.001
Residual disease	3.947	2.030–7.672	<0.001	2.709	1.244–2.608	<0.001
VTE	2.801	1.669–4.717	<0.001	2.155	1.377–3.367	0.001

Abbreviations: VTE, venous thromboembolism; HR, hazard ratio; CI, confidence interval.

## Discussion

Based on our data, 14.5% of the patients with ovarian CCC developed VTE. The reported incidence in the literature ranges from 18.6% to 42% [6,7,10,11]. One possible explanation for the discrepancy between our study and those from Western countries might be racial differences, as Asian peoples have a lower risk of developing VTE than do Caucasians [3]. On the other hand, VTE events might have been missed in the institutional database, leading to an under-estimation of the true incidence. Clinically asymptomatic episodes and events that occurred during follow-up visits in outside hospital can be considered cases in point.

Consistent with the existing literature our results confirm that cases with advanced-stage ovarian CCC were more likely to develop VTE, and have an increased risk for PE [6,8,10,16]. Our study also showed that 6.6% of patients with advanced-stage disease had a thrombus in their proximal veins (IVC, iliac, femoral and popliteal veins). Thus, special attention should be paid to patients with advanced-stage ovarian CCC concerning their VTE risks in the clinical setting.

Concerning the timing of the VTEs, in our study, 36.4% of the clinical VTEs were diagnosed as initial presentations before cancer diagnosis; 12.1%, post-operation; 18.2%, during primary chemotherapy; and 33.3%, at the time of recurrence or progression. As reported by Satoh et al., subclinical VTE was observed in relatively large subsets of ovarian cancer patients prior to primary surgery [17]. However, the routine examination of VTE in patients highly suspicious of ovarian malignancy was not performed in our institution due to concerns with its cost-effectiveness. Still, we can see that 5.3% (12/227) of the patients in this cohort presented with VTE events as their initial symptom. On the other hand, for women with idiopathic VTE and pelvic mass simultaneously, the possibility of gynecologic malignancy should considered in the differential diagnosis. Overall, 12.1% of the VTE events occurred after an operation in spite of peri-operative VTE prophylaxis, and 18.2% of the VTE occurred during primary chemotherapy. Active cancer treatment is strongly associated with VTE, and chemotherapy is a well-recognized risk factor for VTE in cancer patients [2]. Nevertheless, issues of extended prophylaxis and thromboprophylaxis in patients receiving chemotherapy remain controversial [2].

We noticed there were no significant differences between the two groups in terms of age at diagnosis, pre-operative serum CA 125 level, tumor size or ascites volume. Interestingly, fewer patients in the VTE group underwent lymph node dissections in our study. The role of lymphadenectomy in the management of epithelial ovarian carcinoma has been controversial [18]. Lymphadenectomy mainly serves the purpose of staging. No consensus has ever been achieved in the prognostic role of lymphadenectomy in ovarian carcinoma [18,19]. Most patients with VTE diagnosed have advanced-stage disease (from laparotomy findings). However, lymphadenectomy can damage the epithelium of blood vessels, resulting in or promoting the occurrence of VTE. Therefore, it should be individualized whether to perform lymphadenectomy or not.

Our results showed that ovarian CCC patients with VTE had relatively poor survival outcomes, which is consistent with data from previous studies [6,7]. These patients had shorter median PFS and OS compared to patients without VTE. However, further analysis based on VTE timing and type revealed that there was no significant difference in the subgroups. After univariate and multivariate analysis, VTE was confirmed as an independent prognostic factor for both OS and PFS.

When interpreting the data of this study, several points should be noted. First, despite the fact that the database was set up and maintained by research faculty members in our department, the reliability of the database is not clear to the readers and might represent a potential pitfall. Second, the timing distribution of the VTEs was arbitrary. The post-operative period and primary treatment were specifically listed as distinct from ‘during treatment’ due to their differing clinical implications. The existing clinical guidelines recommend VTE prophylaxis for cancer patients through hospitalization after surgery [3,20]. However, extended prophylaxis during adjuvant chemotherapy is still controversial. In our study, a small peak in incidence was observed during chemotherapy, which is notable because patients in our hospital and in other institutions in China continue to receive post-operative chemotherapy in the outpatient setting.

## Conclusion

Clinically symptomatic VTE events occurred in 14.5% of patients with ovarian CCC. Two incidence peaks were noted at disease presentation (36.4%) and at the time of recurrence (33.3%), followed by an adjuvant chemotherapy period (18.2%). Therefore, we suggest that the routine evaluation of VTE is a very important part of the work-up in patients with a high suspicion of ovarian carcinoma. VTE had a negative impact on patient survival.
